# Reliability of Body Temperature Measurements Obtained with Contactless Infrared Point Thermometers Commonly Used during the COVID-19 Pandemic

**DOI:** 10.3390/s21113794

**Published:** 2021-05-30

**Authors:** Filippo Piccinini, Giovanni Martinelli, Antonella Carbonaro

**Affiliations:** 1 IRCCS Istituto Romagnolo per lo Studio dei Tumori (IRST) “Dino Amadori”, 47014 Meldola, Italy; giovanni.martinelli@irst.emr.it; 2 Department of Computer Science and Engineering (DISI), University of Bologna, 40126 Bologna, Italy; antonella.carbonaro@unibo.it

**Keywords:** sensors for medical applications, body temperature, infrared devices, triage emergency rooms, COVID-19

## Abstract

During the COVID-19 pandemic, there has been a significant increase in the use of non-contact infrared devices for screening the body temperatures of people at the entrances of hospitals, airports, train stations, churches, schools, shops, sports centres, offices, and public places in general. The strong correlation between a high body temperature and SARS-CoV-2 infection has motivated the governments of several countries to restrict access to public indoor places simply based on a person’s body temperature. Negating/allowing entrance to a public place can have a strong impact on people. For example, a cancer patient could be refused access to a cancer centre because of an incorrect high temperature measurement. On the other hand, underestimating an individual’s body temperature may allow infected patients to enter indoor public places where it is much easier for the virus to spread to other people. Accordingly, during the COVID-19 pandemic, the reliability of body temperature measurements has become fundamental. In particular, a debated issue is the reliability of remote temperature measurements, especially when these are aimed at identifying in a quick and reliable way infected subjects. Working distance, body–device angle, and light conditions and many other metrological and subjective issues significantly affect the data acquired via common contactless infrared point thermometers, making the acquisition of reliable measurements at the entrance to public places a challenging task. The main objective of this work is to sensitize the community to the typical incorrect uses of infrared point thermometers, as well as the resulting drifts in measurements of body temperature. Using several commercial contactless infrared point thermometers, we performed four different experiments to simulate common scenarios in a triage emergency room. In the first experiment, we acquired several measurements for each thermometer without measuring the working distance or angle of inclination to show that, for some instruments, the values obtained can differ by 1 °C. In the second and third experiments, we analysed the impacts of the working distance and angle of inclination of the thermometers, respectively, to prove that only a few cm/degrees can cause drifts higher than 1 °C. Finally, in the fourth experiment, we showed that the light in the environment can also cause changes in temperature up to 0.5 °C. Ultimately, in this study, we quantitatively demonstrated that the working distance, angle of inclination, and light conditions can strongly impact temperature measurements, which could invalidate the screening results.

## 1. Introduction

Coronavirus disease-2019 (COVID-19) is the most significant illness in recent years. This disease is characterized by high transmissibility and significant morbidity and mortality. After the first officially reported case in Wuhan, China, in December 2019 [1], severe acute respiratory syndrome coronavirus 2 (SARS-CoV-2) became a global phenomenon in only a few months. In March 2020, over 200 countries had reported SARS-CoV-2 cases, and the World Health Organization (WHO) officially declared the COVID-19 outbreak to be a pandemic [2].

COVID-19-related deaths are associated with several factors, primarily including advanced age, diabetes, severe asthma, and various other medical conditions. Older individuals, particularly those over 50 years old, are at high risk of contracting COVID-19 and have a poor prognosis compared to those from younger age groups, possibly due to the patho/physiological changes associated with aging. As a result, the mortality rate tends to be higher among the older population [3]. Furthermore, while men and women have the same prevalence of the disease, men with COVID-19 are at greater risk of suffering worse outcomes and death, independent of age [4]. In addition, compared with those of a white ethnicity, black and south Asian individuals are at greater risk of death [5]. Frequent symptoms include cough, sore throat, diarrhoea, headache, muscle/joint pain, fatigue, and loss of smell and taste [6]. However, the most common symptom is fever (i.e., a body temperature >37.5 °C). Accordingly, temperature is used as a key parameter to determine whether patients might have COVID-19 [7]. For instance, in the four-class system created by the Daegu Medical Association for the rapid classification of patients with COVID-19, patients are termed asymptomatic if they are COVID-19-infected but have a body temperature <37.5 °C; mild if they have a body temperature ≥37.5 °C, but O_2_ supply is not required; moderate if their body temperature is ≥37.5 °C, and O_2_ supply is required; and severe if the body temperature is ≥38 °C, and high-flow O_2_ supply or mechanical ventilation is needed [8].

The correlation between high temperature and COVID-19 infection is so strong that several governments have imposed screening protocols involving a body temperature check at the entrances to hospitals, airports, train stations, churches, schools, shops, sports centres, offices, etc. [9]. From a practical standpoint, emergency triage rooms and general checkpoints have been established at the entrances to most crowded places, and, in the event that a person is identified to have a high temperature, access is denied to reduce the risk of transmitting the virus [10]. For this reason, contactless devices to measure body temperature have rapidly become popular all over the world [11].

Among the different systems available for temperature screenings, the most widely used are contactless infrared cameras and point thermometers [12]. These systems measure the infrared radiation emitted by different bodies. Infrared cameras use a grid of thermal detectors and can be used to construct a 2D temperature map. Instead, contactless infrared point thermometers use a single thermal detector per device and, accordingly, give a single temperature value as their output. A physical body with a temperature above absolute zero (i.e., –273.15 °C; 0 Kelvin) emits electromagnetic radiation proportional to its intrinsic body temperature. Infrared radiation is a part of this intrinsic temperature. Technically, infrared radiation covers a portion of the whole range of the electromagnetic spectrum, starting at the visible range of about 0.78 μm and ending at wavelengths of approximately 1 mm. In general, thermal detectors are broadband, meaning that they collect all infrared radiation from the source. However, wavelengths ranging from 3 to 14 μm are typically used for measuring body temperature. Recently, Chen et al. mathematically described how body temperature can be measured using a contactless infrared point-thermometer [13]. Briefly, infrared radiation is emitted from the body surface and penetrates the atmosphere. With the help of a lens (i.e., input optics), the radiation beam is focused onto a detector that generates an electrical signal proportional to the input radiation. Thanks to this phenomenon, contactless infrared point thermometers are commonly used for measuring body temperature in a rapid, non-destructive, non-interactive, and non-invasive manner. Contactless infrared point thermometers are generally composed of a lens, a detector, a signal amplifier, a source of digital signal processing, and a display. Today, the market offers a wide range of contactless infrared point thermometers, sometimes called infrared contactless thermal guns [14]. In general, these devices are fairly inexpensive (typically less than €100) and do not differ significantly in terms of hardware and software. Most commonly, 2 °C is reported in the relevant datasheets as being generally accurate within a range of 36–39 °C. Furthermore, these devices can be used to measure body temperature at different body sites [15]. However, the forehead is the most typical site of measurement [16].

During the COVID-19 pandemic, there has been a substantial increase in the availability of different models of non-contact infrared point thermometers and in courses for teaching operators how to use and maintain these instruments. From a practical viewpoint, in a very short space of time, all shops, workplaces, and public buildings employed operators mainly equipped with contactless infrared devices to screen people at entrances by measuring their body temperatures, thus reducing the risk of viral transmission through asymptomatic carriers [17]. Although such a practice incurs substantial costs, the key issue here is not the expense but the reliability of the measurements, as misclassifying healthy people or missing infected individuals can have serious consequences [18]. For example, a cancer patient going to a hospital for treatment could be denied entry due to a erroneous high temperature measured at the entrance [19]. On the other hand, an infected individual with an underestimated temperature reading could have a serious health impact if allowed into a crowded public place such as a supermarket [20]. Previous studies reported metrological experiments to evaluate the performance of contactless infrared point thermometers using tuneable artificial heat sources. For instance, Fletcher et al. [21] recently analysed three non-contact infrared point thermometers using blackbody sources with temperature determined using calibrated platinum resistance thermometers. Based on analyses performed in the laboratory using these artificial heat sources, the authors concluded that two out of the three devices suffered from large measurement errors falling far outside the accuracy range stated by their manufacturers, as well as the medical standard to which these devices are intended to adhere. Finally, in a very recent paper, Dell’Isola et al. [22] carefully analysed the effects of many metrological and subjective issues on the reliability of the body temperature measurement. They clarified that the body temperature measurement is influenced by the unavoidable instrumental uncertainties and by the operator’s ability, but also by numerous other quantities such as: (a) the emissivity and the reflection coefficient of the emitting skin surface; (b) the transmission coefficient of the medium between the sensor and the target; (c) the average radiant temperature of the measurement environment (i.e., the reflected temperature); (d) the distance and consequent size of the target (effect of the size of the source). In addition, they explained how the measurement is affected by the intrinsic complexity and variability of the subjective measurand and to the homeostatic mechanisms of body thermoregulation, mainly under the hypothalamic control and conditioned by (I) several individual factors (e.g., comorbidities, age, physical activity, digestion, stress, use of drugs and smoking); (II) temporal variables (e.g., circadian rhythm, menstrual cycle); (III) spatial variables (e.g., body and skin); (IV) environmental conditions (e.g., indoor/outdoor). They also provided illustrations to show the (i) root of the causes of noncontact temperature measurement uncertainty; (ii) body temperature variability at different body sites; (iii) body temperature variability after meals. Finally, they proposed a two-step screening decision protocol to better prevent the spread of COVID-19. The protocol takes into account both the traditional instrumental uncertainty sources and clinical–medical ones related to the subjectivity of the measurand. The first step is based on a deterministic temperature threshold (generally set at 37.5 °C to avoid a large number of false positives). In this step, the body temperature is estimated from the forehead using a simple and quick noncontact temperature thermometer. The second step (performed when the first-step noncontact measurement falls within an uncertainty zone) is based on a statistical threshold value determined on the basis of the sample-measured temperatures at real measurement conditions and the adopted procedure. Practically, in the second step, the temperature assessment is performed by means of an axillary contact temperature measurement and after the subject has been at rest to thermally stabilize for at least 15 min in an indoor environment. The availability of data acquired in different real-world scenarios to assess the de facto reliability of non-contact infrared point thermometers for estimating the body temperature of human subjects, together with the extensive analysis provided by Dell’Isola et al. and the designed protocol, may represent a great help to support national authorities to better set up the obligation to measure body temperature for limiting the risk of contagion.

In the present study, we collected and then shared quantitative data under several real scenarios to highlight the most typical incorrect uses of infrared point thermometers and the resulting drifts in measurements of body temperature. In particular, we tested four different commercial contactless infrared point thermometers used on a daily basis at our cancer institute (IRST, Meldola, Italy) and performed five different tests to report the de facto: (a) intra- and (b) inter-rater reliability, as well as the dependence of the measurements on the (c) working distance, (d) angle of inclination of the device, and (e) spurious infrared radiation (i.e., ambient radiation). We considered representative problems affecting the real measurements taken in an emergency triage room and at a temperature checkpoint, as the operators performing this task typically do not use a ruler or goniometer and visually evaluate the forehead–device distance when acquiring the measurement.

The quantitative data obtained from the present experiments show that even a slightly incorrect use of contactless infrared point thermometers can lead to substantial inaccuracies in the measurement of body temperature. Our data confirm that several factors can invalidate temperature screening. This is important information for temperature screening operators and for organisations planning temperature checks at the entrances of stores, workplaces, and public buildings. In general, it is recommended that the body temperature be measured a second time after individuals have become acclimatized to being indoors [19]. However, a better understanding of the de facto limits of non-contact infrared devices among the devices’ operators and the individuals being screened could help obtain more reliable data to protect people from the spread of SARS-CoV-2 infection during this difficult time.

## 2. Materials and Methods

### 2.1. Devices Used in the Experiments

**Figure 1:**
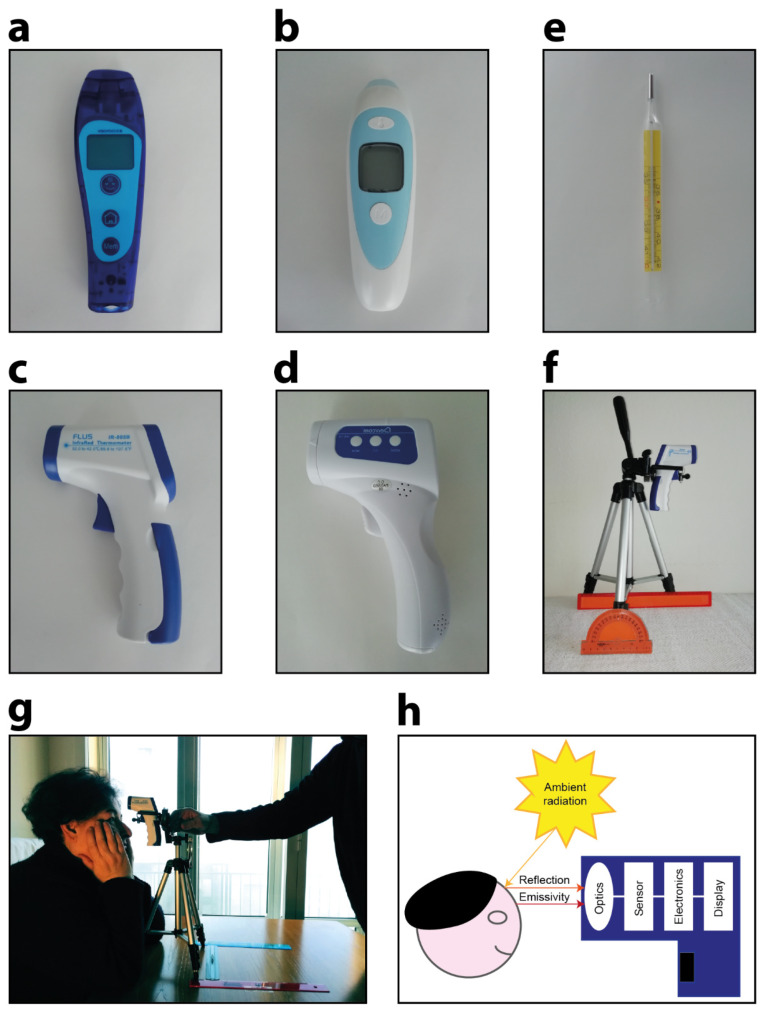
Contactless infrared thermometers used in this work: (**a**) TECNIMED VisioFocus PRO 06480; (**b**) Medek MDI261; (**c**) FLUS IR-805B; (**d**) Berrcom JXB-178 (T2). (**e**) Axillary mercury-based analogical thermometer. (**f**) Ruler, goniometer, and tripod used in the experiments. (**g**) Representative photograph acquired during the experiments. (**h**) Schematic representation of the detected infrared radiation composed of the emitted signal and the component of the ambient radiation reflected from the surface of the body of the person.

In the experiments, we simulated an operator stationed at the entrance to a public place to assess the body temperatures of people interested in accessing the building. Four different commercial models of non-contact infrared point thermometers used daily in our cancer centre (IRST, Meldola, Italy) were tested in the experiments:

- Company: TECNIMED (Varese, Italy), model: VisioFocus PRO 06480; working distance automatically suggested in real-time (approximately 5–10 cm; Figure 1a);
- Company: Medek (Shenzhen, China), model: MDI261; working distance reported in the datasheet: 1–3 cm (Figure 1b);
- Company: FLUS (Shenzhen, China), model: IR-805B; working distance reported in the datasheet: 5 cm (Figure 1c);
- Company: Berrcom (Guangdong, Cina), model: JXB-178; working distance reported in the datasheet: 3–5 cm (Figure 1d).

Table 1 lists the main manufacturers’ technical specifications reported in the datasheets of the four non-contact infrared point thermometers considered in this study. Hereafter, the four thermometers will be referenced using the acronym “T*n*”, with *n* = 1, 2, 3, or 4. However, as our analysis was designed to formulate general considerations rather than to validate a specific device, we will not specify which device is being referred to as T1, T2, T3, or T4 to avoid commercial issues when reporting the quantitative data.

All the measurements were acquired in the same room under the same light conditions, except when explicitly reported. The temperature and humidity of the room were monitored using a BAR208SX Weather Station (Oregon Scientific, Tualatin, OR, USA). Notably, these data were not considered in the quantitative analysis performed in this study. However, both are always directly reported in the Appendix A Table A1, Table A2, Table A3, Table A4, Table A5, Table A6, Table A7, Table A8, Table A9, Table A10, Table A11 and Table A12, should future studies require them. To acquire a reference temperature, we used a classic standard axillary mercury-based analogical thermometer for measuring body temperature from the armpit (Figure 1e). Five min was the time required for each measurement with this analogical thermometer. Before each round of data acquisition, three measurements were acquired from the right armpits of the operators, and the average value was then considered as the ground truth (GT). All measurements were acquired from the foreheads of three healthy subjects: Subject 1 was a 65-year-old woman, Subject 2 was a 65-year-old man, and Subject 3 was a 35-year-old man. These measurements simulated the person to be assessed in an emergency triage room. Surrounding materials can influence the temperature measurements of self-heating objects [23]. Thus, during the acquisitions, we carefully ensured that no sweat and/or make-up was present on the foreheads of the subjects. Notably, all the subjects involved in the experiments simply volunteered to test the infrared devices; no personal sensitive health data were shared in this work. However, to avoid potential disclosures, all 3 subjects involved in the experiments gave written informed consent to publicly authorize, without restriction, the reproduction, treatment, and analysis of the collected data. To acquire the measurements we used a ruler, a goniometer, and a tripod (Figure 1f). We then asked Subject 1/Subject 2/Subject 3 to sit down, tilt up their heads with their arms resting on the table, and avoid moving (Figure 1g).

### 2.2. Inter- and Intra-Rater Reliability Experiment: Description

Here, we define the terms inter-rater reliability and intra-rater reliability, also known as inter- and intra-observer variability or reproducibility and repeatability, respectively [24]. Inter-rater reliability (i.e., inter-observer variability or reproducibility) is defined as the closeness of the agreement between measurements of the same object, carried out under modified measurement conditions; Intra-rater reliability (i.e., intra-observer variability or repeatability) is the closeness of the agreement between successive measurements of the same object, carried out under the same measurement conditions. In our experiments, the object was the body temperature of Subject 1/Subject 2/Subject 3, and the modified condition was the contactless infrared thermometer used.

To keep the experimental settings constant, the shutters on the windows in the room were closed, and the room was illuminated by a constant artificial light. To avoid fluctuations in body temperature, the temperature of Subject 1/Subject 2/Subject 3 was first measured using a traditional axillary mercury-based analogical thermometer and then measured again shortly afterwards with each of the non-contact infrared point thermometers. The body temperature of a person varies throughout the day, with lower temperatures generally observed in the morning and higher temperatures in the late afternoon/evening. Accordingly, we acquired the measurements over a short time period, and a restricted number of repetitions were carried out to limit the normal fluctuations in body temperature. Specifically, 10 measurements for each thermometer were acquired without measuring the precise working distance and angle of inclination between the forehead and the thermometers, except *i* perpendicular to the forehead. The experiment was repeated a total of three times for each subject. To better simulate real-life temperature acquisition in a public setting, we decided to rotate the devices, always acquiring only one measurement at a time with each to ensure a slightly different distance and angle between the measurements acquired using the same thermometer.

### 2.3. Body—Device Distance Experiment: Description

**Figure 2:**
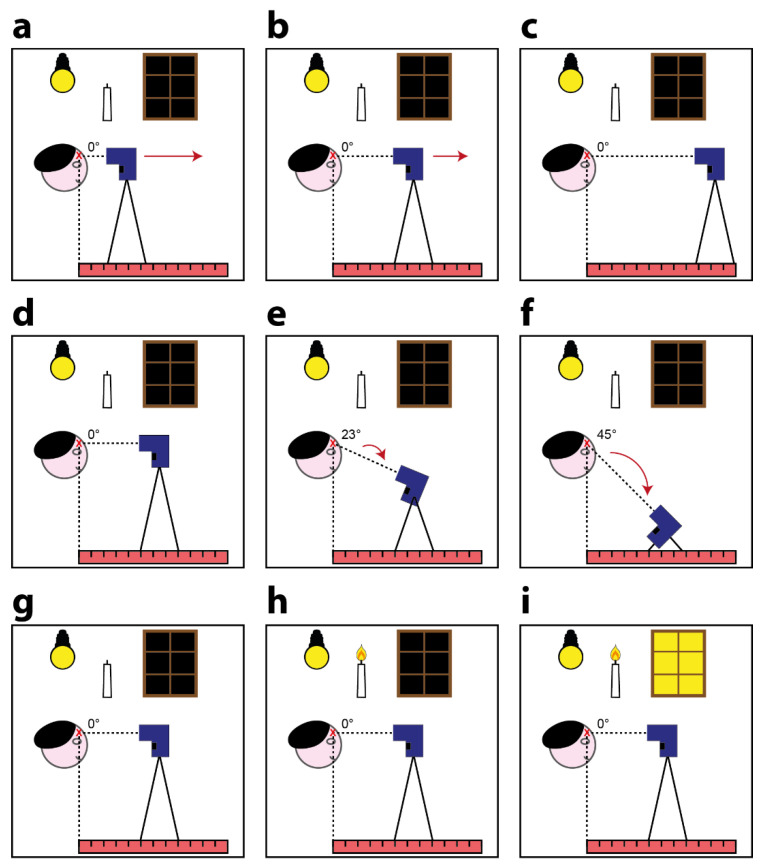
Schematic representation of the performed experiments: (**a**–**c**) body–device distance experiment: While keeping the light and the body–device angle conditions constant, we modified the distance of acquisition; (**d**–**f**) body–device angle experiment: while keeping the light and the body–device distance conditions constant, we modified the angle of acquisition; (**g**–**i**) light influence experiment: while keeping the body–device distance and the body–device angle conditions constant, we modified the illumination conditions during the acquisitions.

The majority of contactless infrared point thermometers have a fixed focus. From a practical point of view, the lens focuses at a specific working distance. This means that before acquiring the measurement, the operator performing the task must ensure that the distance between the person’s forehead and the device is within the range reported in the datasheet of the device. Usually, it remains possible to use the device to measure outside the optimal measuring range. However, from a logical standpoint, the reliability of the measurements will be lower in such areas. To assess the extent to which the working distance (i.e., the distance between the forehead and the thermometer) affects the measurement of body temperature, we maintained the thermometer perpendicular to the forehead and, using a goniometer, acquired five measurements for each device in 10 different positions (Figure 2a–c). For all the devices, we acquired measurements while increasing the body–device distance from 1 to 10 cm, in 1 cm increments. The shutters of the windows in the room were closed, and the room was illuminated only by a constant artificial light. We asked Subject 1/Subject 2/Subject 3 to sit down, tilt up their heads with their arms resting on the table, and avoid moving. Then, we secured the thermometer on a tripod and, using a ruler, measured the distance between the Subject’s forehead and the terminal part of the thermometer. Before moving the tripod, we acquired five measurements for each position. We then exchanged the thermometer and repeated the acquisitions.

### 2.4. Body—Device Angle Experiment: Description

During real-life data acquisition, the angle of inclination of the contactless infrared thermometer (i.e., the angle between the line perpendicular to the forehead and the normal straight line from the thermometer) is often not optimal (i.e., 0 degrees). This is because the measurement is typically acquired while both the person being assessed and the operator are in standing positions, and the difference between the heights of the two people determines an angle of inclination. For example, an adult operator may need to measure the body temperature of a young child, and a short operator may need to measure the temperature of a tall subject at the forehead. To assess the degree to which the angle of inclination affects the measurement of body temperature based on the different datasheets, we maintained the TECNIMED VisioFocus PRO 06480 at 7 cm, the Medek MDI261 at 3 cm, and the other two thermometers at 5 cm, acquiring five measurements for each device at three different angles: 0 degrees (i.e., with the thermometer perpendicular to the forehead; Figure 2d), 23 degrees (Figure 2e), and 45 degrees (Figure 2f). For the other experiments, the shutters of the windows in the room were closed, and the room was illuminated only by a constant artificial light. Again, we asked *Subject 1*/*Subject 2/Subject 3* to sit down, tilt up their heads with their arms resting on the table, and avoid moving. We then secured the thermometer on the tripod and, using a goniometer, measured the inclination angle between the *Subject*’s forehead and the head of the thermometer, moving the tripod down so it always pointed to the middle of the forehead. Before changing the angle of the tripod, we acquired five measurements for each inclination. We then exchanged the thermometer and repeated the acquisitions.

### 2.5. Light Influence Experiment: Description

In general, the radiation measured by a contactless infrared thermometer can be considered as the sum of two main components (Figure 1h): the infrared radiation emitted by the body of the person and the portion of ambient infrared radiation reflected by the body surface that enters the thermometer’s components (i.e., the lens, sensor, signal amplifier, and digital signal processor). To assess the extent to which different light conditions (i.e., light in the environment) affect the measurement of body temperature, we positioned the thermometers perpendicular to the foreheads of *Subject 1*/*Subject 2/Subject 3* and maintained the TECNIMED VisioFocus PRO 06480 at 7 cm, the Medek MDI261 at 3 cm, and the other two thermometers at 5 cm. We then acquired five measurements for each device under three different light conditions:

1) With the shutters of the windows in the room closed and the room illuminated only by a constant artificial light (i.e., the same conditions as those in the other experiments, Figure 2g). This condition aimed to simulate real-life acquisitions performed, for instance, in an emergency triage room during the late afternoon/evening.
2) With the shutters of the windows in the room closed and the room illuminated only by constant artificial light and a small candle (Figure 2h). This condition again aimed to simulate real-life acquisitions performed in an emergency triage room during the late afternoon/evening but with a different source of light than that of the first condition.
3) With the shutters of the windows in the room kept fully open to allow sunlight to illuminate the room. The artificial light was retained, and the candle remained lit (Figure 2i). This condition aimed to simulate real-life acquisitions performed, for instance, in an emergency triage room in the morning/early-afternoon. We performed these experiments only on sunny days to ensure a significant difference compared to the other conditions featuring closed window shutters. However, the acquisitions were performed while ensuring that the rays of the sun did not touch the subjects’ foreheads.

As in the other experiments, we asked *Subject 1*/*Subject 2/Subject 3* to sit down, tilt up their heads with their arms resting on the table, and avoid moving. We then secured the thermometer with a tripod and, using a ruler and goniometer, measured the working distance and inclination angle. We next modified the light settings before exchanging the device.

## 3. Results

### 3.1. Inter- and Intra-Rater Reliability Experiment: Results

**Figure 3:**
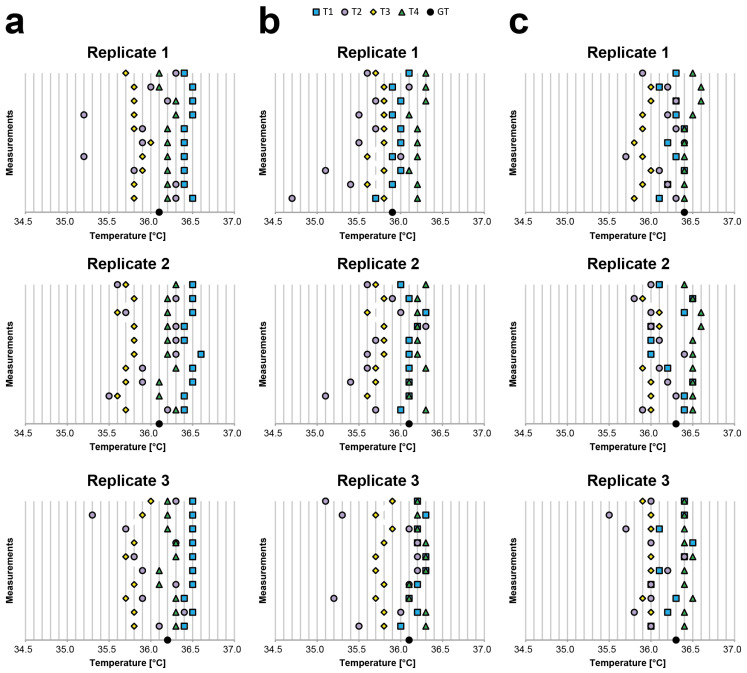
Visual representation of the temperature data (reported on the *x*-axis in °C) collected during the inter- and intra-rater reliability experiment. For each replicate, 10 measurements for each thermometer were acquired while always acquiring only one measurement at a time for each to ensure a slightly different distance and angle between the measurements acquired using the same thermometer; (**a**) Subject 1, (**b**) Subject 2, and (**c**) Subject 3 values.

Table A1, Table A2 and Table A3 present the values obtained by analysing the body temperatures of Subject 1, Subject 2, and Subject 3, respectively (reported in °C). The same values are also visually represented on the graphs in Figure 3. Notably, in this experiment, (a) the body–device distance and angle were visually evaluated to simulate acquisitions under real-life settings, and (b) we did not filter the data to present the de facto expected values, especially when a single measurement is acquired. All the values use °C, except when specifically reported otherwise.

For the inter-rater reliability (i.e., inter-observer variability/reproducibility), we evaluated the difference between the average values and GT using a classic axillary mercury-based analogical thermometer. For Subject 1, the average difference with respect to GT was −0.33 for T1, 0.16 for T2, 0.35 for T3, and −0.08 for T4; for Subject 2, −0.05 for T1, 0.36 for T2, 0.28 for T3, and −0.18 for T4; and for Subject 3, 0.08 for T1, 0.27 for T2, 0.37 for T3, and −0.13 for T4. These differences become substantial when considering the difference between the average values of two different thermometers. For Subject 1, the worst cases were always related to T1 and T3 (differences: 0.61, 0.76, and 0.67). For Subject 2, the worst cases were related to the T4 and T2/T3 (differences: T4-T2, 0.68; T4-T2, 0.52; and T4-T3, 0.45). For Subject 3, the worst cases were related to T4 and T2/T3 (differences: T4-T3, 0.55; T4-T3, 0.51; and T4-T2, 0.46). These values prove that it is simple to find a difference greater than 0.5 °C. Accordingly, it is very difficult to determine the real body temperature of a person using a random contactless infrared thermometer when the operator visually estimates the body–device distance and angle.

For the intra-rater reliability (i.e., intra-observer variability/repeatability), we evaluated the average differences between the maximum (i.e., Max) and minimum (i.e., Min) values obtained for each device. The average Max-Min differences for Subject 1 were: 0.13 for T1, 1.00 for T2, 0.27 for T3, and 0.20 for T4; for Subject 2, 0.33 for T1, 1.23 for T2, 0.20 for T3, and 0.20 for T4; and for Subject 3, 0.43 for T1, 0.73 for T2, 0.16 for T3, and 0.16 for T4. This shows that half of the devices provided significantly different measurements with a de facto difference higher than 0.2 °C, as declared in the datasheet, when used to acquire the temperature from the same person multiple times by visually evaluating the body–device distance and angle.

### 3.2. Body—Device Distance Experiment: Results

**Figure 4:**
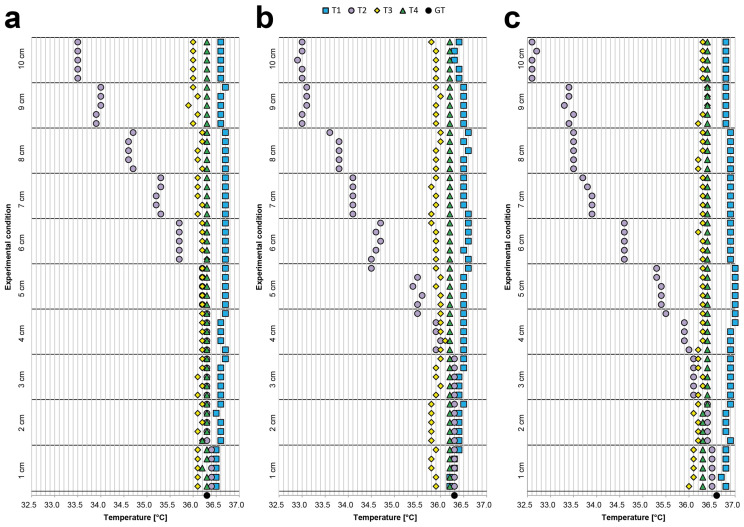
Visual representation of the temperature data (reported on the *x*-axis in °C) collected during the body–device distance experiment. We maintained the thermometer perpendicular to the forehead and, using a goniometer, acquired 5 measurements for each device in 10 different positions; (**a**) Subject1, (**b**) Subject2, and (**c**) Subject3 values.

Table A4, Table A5 and Table A6 provide the values obtained by analysing the body temperatures of Subject 1, Subject 2, and Subject 3, respectively (reported in °C). The same values are also visually represented in the graphs provided in Figure 4. Notably, during this experiment, (a) the body–device distance was modified to assess the corresponding fluctuations in measurements; moreover, (b) we did not filter the data to show the de facto expected values, especially when a single measurement was acquired. Furthermore, we again acquired measurements at 10 different distances within a range of only 10 cm to illustrate that even a small difference in position can strongly affect the measurement. All values are in °C, except when specifically reported otherwise.

We evaluated the difference between the Max and Min values obtained for each device. For Subject 1, the Max–Min differences were 0.2 for T1, 2.9 for T2, 0.4 for T3, and 0.1 for T4; for Subject 2, 0.4 for T1, 3.4 for T2, 0.3 for T3, and 0.0 for T4; and for Subject 3, 0.3 for T1, 3.9 for T2, 0.4 for T3, and 0.1 for T4. These values show that, for half of the devices, a difference of just a few centimetres can yield large differences in body temperature measurements.

Finally, we compared the average temperatures measured with the different thermometers. For Subject 1, the smallest difference between the average values was 0.17 (represented by the difference between T4 and T3), while the greatest difference was 1.18 (i.e., between T1 and T2). For Subject 2, the smallest difference was 0.27 (represented by the difference between T1 and T4), and the greatest difference was 1.61 (i.e., between T1 and T2). For Subject 3, the smallest difference was 0.12 (represented by the difference between T2 and T3), and the greatest difference was 2.06 (i.e., between T1 and T2). Once again, these values show that the measurements obtained using a contactless infrared point-thermometer can be highly unreliable when the operator visually estimates the body–device distance.

### 3.3. Body—Device Angle Experiment: Results

**Figure 5:**
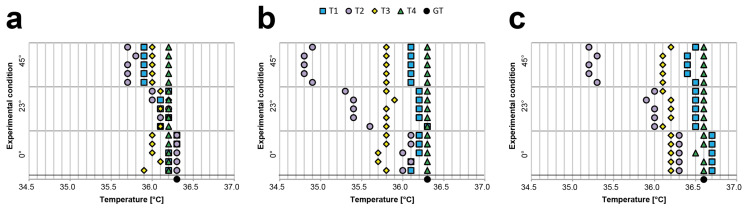
Visual representation the temperature data (reported in the *x*-axis in °C) collected during the body–device angle experiment. We kept the TECNIMED VisioFocus PRO 06480 at 7 cm, the Medek MDI261 at 3 cm and the other two thermometers at 5 cm, and we acquired five measurements for each device at three different angles: 0 degrees (i.e., thermometer perpendicular to the forehead), 23 degrees, and 45 degrees; (**a**) *Subject* *1*, (**b**) *Subject* *2*, and (**c**) *Subject* *3* values.

Table A7, Table A8 and Table A9 provide the values in °C obtained by analysing the body temperatures of Subject 1, Subject 2, and Subject 3, respectively. The same values are also visually represented in the graphs in Figure 5. Notably, during this experiment, (a) the body–device angle was modified (i.e., by 0, 23, and 45 degrees) to assess the corresponding fluctuations in measurements; moreover, (b) we did not filter the data to show the de facto expected values, especially when a single measurement was acquired. Furthermore, we acquired measurements at angles that were not extremely sharp to simulate real-life scenarios (e.g., when the person to be assessed is a child). All values are in °C, except when specifically reported otherwise.

We evaluated the differences between the Max and Min values obtained for each device. For Subject 1, the Max–Min differences were 0.4 for T1, 0.6 for T2, 0.3 for T3, and 0.0 for T4; for Subject 2, 0.2 for T1, 1.3 for T2, 0.2 for T3, and 0.0 for T4; and for Subject 3, 0.3 for T1, 1.1 for T2, 0.1 for T3, and 0.1 for T4. These values show that, for half of the devices, the measurements acquired were highly sensitive to the angle of measurement. This was expected because maintaining the device at the same distance from the forehead while adjusting the z-position to acquire a measurement from the same part of the forehead at a wider angle of inclination creates a larger normal distance between the terminal point of the device and the surface of the forehead. Accordingly, the fluctuations in the measurements should logically be similar to those observed in the previous body–device distance experiment. Again, we compared the average temperatures measured with the different thermometers. For Subject 1, the differences were always lower than 0.2. However, the difference between T4 and T2 for Subject 2 was 0.86 and that for Subject 3 was 0.75, proving again that when the operator visually estimates the body–device angle, the measurements obtained may be unreliable.

### 3.4. Light Influence Experiment: Results

**Figure 6:**
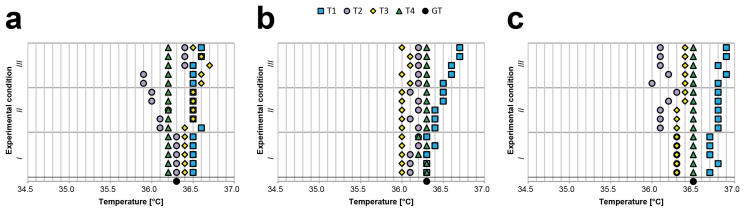
Visual representation of the temperature data (reported on the *x*-axis in °C) collected during the light influence experiment. We positioned the thermometers perpendicular to the forehead of Subject 1/Subject 2/Subject 3 and maintained the TECNIMED VisioFocus PRO 06480 at a 7 cm distance, the Medek MDI261 at a 3 cm distances, and the other two thermometers at 5 cm distances. We then acquired five measurements for each device under three different conditions of light (i.e., *I*, *II*, *III*, as described in the text); (**a**) Subject 1, (**b**) Subject 2, and (**c**) Subject 3 values.

Table A10, Table A11 and Table A12 provide the values in °C, which were obtained by analysing the body temperatures of Subject 1, Subject 2, and Subject 3, respectively. The same values are also visually represented in the graphs provided in Figure 6. 

Notably, during this experiment: (a) the light conditions were modified to assess the corresponding fluctuations in measurements; moreover, (b) we did not filter the data to show the de facto expected values, especially when a single measurement was acquired. All values are in °C, except when specifically reported otherwise.

We evaluated the differences between the Max and Min values obtained for each device. For Subject 1, the Max–Min differences were 0.1 for T1, 0.5 for T2, 0.3 for T3, and 0.0 for T4; for Subject 2, 0.4 for T1, 0.1 for T2, 0.1 for T3, and 0.1 for T4; and for Subject 3, 0.2 for T1, 0.3 for T2, 0.1 for T3, and 0.0 for T4. These values prove that half of the devices were sensitive to light conditions and that the acquisition time (e.g., day or evening) can significantly affect the measurement of body temperature.

Again, we compared the average temperatures measured with the different thermometers. For Subject 1, the greatest difference between the average values was 0.33 (between T1 and T2); the greatest difference for Subject 2 was 0.44 (i.e., between T1 and T3), and the greatest difference for Subject 3 was 0.60 (i.e., between T1 and T2). These values once again indicate that determining the real body temperature of a person using a random contactless infrared thermometer is challenging and that the acquisition settings and light conditions affect the measurement.

## 4. Discussion

The main goal of this work was to evaluate the reliability of obtaining body temperature measurements using contactless infrared point thermometers to highlight: (a) the common sources of errors when using these devices and (b) the reliability that operators using this technology should expect.

In the first test, we acquired 30 measurements from the same subjects to check whether the de facto difference between the measurements was <= 0.2 °C, as reported by the manufacturers in the datasheet. We showed that half of the devices tested were characterised by repeatedly falling out of this range. Then, we computed the average temperature acquired using different thermometers and showed that when comparing two of these devices, it was easy to find a difference even higher than 0.5 °C. Next, we performed three different experiments to determine the consequences of typical issues when these devices are used for real temperature screenings. First, we gradually modified the forehead–thermometer distance to analyse the changes in measurements when the operator acquires the temperature more than once but always visually evaluates the working distance. Then, while maintaining the same distance, we modified the angle of inclination of the devices to simulate an adult operator seeking to measure the temperature of a child and a short operator seeking to measure the temperature of a tall subject. Finally, we modified the light conditions to show that the measurements obtained using this technology are also affected by the light in the environment and that these devices should not be used in direct sunlight. Notably: (1) all data were reported without data filtering or data processing to show the de facto fluctuations in measurements that operators could expect when using contactless infrared devices, and (2) our experiments were designed for a general analysis of this contactless infrared technology and not for the validation of a specific device. Accordingly, we listed the models of the devices tested in this work, but, when reporting the quantitative data, we simply referred to the devices with anonymous acronyms, without specifying which thermometer the acronym referred to. The results demonstrate that for half of the devices, even a difference of just a few centimetres can result in a very large difference in body temperature measurements. The same results were obtained for the angle of inclination and lighting conditions. One of the limits of this study is that we considered only healthy volunteers. Nevertheless, our results confirm that these devices are generally characterized by a high bias [25]. specifically, we demonstrated that, compared to temperatures acquired using a classic axillary mercury-based analogical thermometer, the temperatures acquired using half of the infrared devices were, on average, higher by 0.4 °C. This shows, again, that the correlation between infrared and axillary thermometers is usually poor, making these devices non-interchangeable [26]. Furthermore, differences of as much as 0.7 °C were observed between two of the contactless infrared point thermometers, highlighting the difficulty in acquiring real body temperatures and suggesting that authorities should approve only devices with validated accuracy for use in population screening.

This analysis appears to invalidate several temperature screening methods typically performed at the entrances to indoor public places, with related costs for the relevant public structures. However, our findings highlight important considerations for operators that could help improve the reliability of contactless infrared point thermometers: (1) It is important to inform the operator of the working distance reported in the device’s datasheet and to emphasize the importance of carefully evaluating the forehead–device distance before acquiring each measurement. (2) It is important to keep the device perfectly perpendicular to the forehead of the adult/child to be assessed. (3) As the light of the environment is an important factor for accuracy, it is better to acquire temperature measurements in indoor areas illuminated by constant artificial lighting. If an operator fails to account for these three factors, the data obtained should be considered only as roughly qualitative results to better understand whether a person has a high fever, rather than accurate quantitative values.

## 5. Conclusions

In this work, we tested several contactless infrared point thermometers in different settings that simulate real-life public scenarios. The analyses in this work are not intended to represent a specific validation of contactless infrared devices but merely to highlight the observation that even the slightly incorrect use of an infrared thermometer can lead to substantially unreliable measurements of body temperature. However, the experiments performed show the following:

1) Acquiring measurements without carefully monitoring the working distance and angle of inclination can lead to a large discrepancy between subsequent body temperature estimations;
2) Light conditions influence the measurements, and, accordingly, the ambient radiation in the assessment room should be monitored, e.g., by using constant artificial lights.

The data obtained in this work can thus support authorities in better organising emergency triage rooms and temperature checkpoints in the following ways:

1) By suggesting that operators use only thermometers from a list of approved devices;
2) By using public information methods (e.g., advertisements) to inform operators about the correct working distance, angle of inclination, and light conditions to obtain more reliable measurements.

The knowledge of these cautions give both operators and the individuals being screened a better understanding of the *de facto* limits of non-contact infrared devices. In addition, our data support the conclusions of Dell’Isola et al. [22] that, to improve the reliability of screenings to prevent the spread of COVID-19 disease, proposed the following:

- To punctually establish the measurement conditions and method;
- To set a fixed temperature threshold reference, by considering an assigned measurement body site;
- To accurately estimate the measurement uncertainty, taking into account the main contributions at the real operative measurement conditions;
- To transpose the threshold reference value as a function of the body site used;

To perform a double-step measurement protocol consisting of (a) a first step, with a noncontact body temperature measurement, and (b) a second step, with a further contact body temperature measurement when the measured value falls within the uncertainty zone.

This procedure would help the relevant authorities to obtain more reliable body temperature data to protect people from the spread of the SARS-CoV-2 virus and future infections.

In conclusion, although our findings show that contactless infrared point thermometers are a highly useful tool to screen the body temperatures of subjects, the data obtained confirm that such thermometers must be used with caution because of their generally high bias. In general, it would be more appropriate to consider these instruments as qualitative devices rather than as a means of accurately determining absolute body temperature. However, waiting for the prospective subject to become acclimatized to being indoors and carefully considering the working distance, angle of inclination, and light conditions may effectively improve the reliability of the measurements to also allow for quantitative considerations.

## Figures and Tables

**Table 1:** Main manufacturer’s technical specifications for the four non-contact infrared point thermometers considered in this study.

Company	TECNIMED	Medek	FLUS	Berrcom
Model	VisioFocus PRO 06480	MDI261	IR-805B	JXB-178
Manufactured in:	Italy	China	China	China
Operating room temperature [°C]	16–40	10–40	10–50	10–40
Measuring range [°C]	34–42.5	32–43	32–42	32–42.9
Resolution [°C]	0.1	0.1	0.1	0.1
Accuracy [°C]	±0.3 from 34 to 35.9 ±0.2 from 36 to 39 ±0.3 from 39.1 to 42.5	±0.3 from 34 to 35.4 ±0.2 from 35.5 to 42 ±0.3 from 42.1 to 42.9	±0.3 from 32 to 35.9 ±0.2 from 36 to 39 ±0.3 from 39.1 to 42	±0.3 from 32 to 34.9 ±0.2 from 35 to 42 ±0.3 from 42.1 to 42.9
Working distance [cm]	5–10	1–3	5	3–5
Standards	ASTM E1965.98	ASTM E1965.98	ASTM E1965.98	ASTM E1965.98

## Data Availability

The authors confirm that all the data supporting the findings of this study are included within the article.

## Appendix

**Table A1:** Intra- and inter-rater reliability experiment. *Subject 1* body temperature data.

**Room temperature [°C]**		19.8		
**Room humidity**		53%		
**Replicate 1**				
**Body temperature GT [°C]**		36.1		
**Number**	**T1 [°C]**	**T2 [°C]**	**T3 [°C]**	**T4 [°C]**
1	36.5	36.3	35.8	36.2
2	36.4	36.3	35.8	36.2
3	36.4	35.8	35.9	36.2
4	36.4	35.2	35.9	36.2
5	36.4	35.9	36.0	36.2
6	36.4	35.9	35.8	36.2
7	36.5	35.2	35.8	36.3
8	36.5	36.2	35.8	36.3
9	36.5	36.0	35.8	36.1
10	36.4	36.3	35.7	36.1
*Min [°C]*	*36.4*	35.2	35.7	36.1
*Max [°C]*	*36.5*	36.3	36.0	36.3
*Max–Min difference [°C]*	*0.1*	1.1	0.3	0.2
*Mean [°C]*	*36.44*	35.91	35.83	36.20
*GT–Mean difference [°C]*	*−0.34*	0.19	0.27	−0.10
**Replicate 2**				
**Body temperature GT [°C]**		36.1		
**Number**	**T1 [°C]**	**T2 [°C]**	**T3 [°C]**	**T4 [°C]**
1	36.4	36.2	35.7	36.3
2	36.4	35.5	35.6	36.1
3	36.5	35.9	35.7	36.1
4	36.5	35.9	35.7	36.3
5	36.6	36.3	35.8	36.2
6	36.4	36.3	35.8	36.2
7	36.4	36.3	35.8	36.2
8	36.5	35.7	35.6	36.2
9	36.5	36.3	35.8	36.2
10	36.5	35.6	35.7	36.3
Min [°C]	36.4	35.5	35.6	36.1
Max [°C]	36.6	36.3	35.8	36.3
Max–Min difference [°C]	0.2	0.8	0.2	0.2
Mean [°C]	36.47	36.00	35.72	36.21
GT–Mean difference [°C]	−0.37	0.10	0.38	−0.11
**Replicate 3**				
**Body temperature GT [°C]**		36.2		
**Number**	**T1 [°C]**	**T2 [°C]**	**T3 [°C]**	**T4 [°C]**
1	36.4	36.1	35.8	36.3
2	36.5	36.4	35.8	36.3
3	36.4	35.9	35.7	36.3
4	36.5	36.3	35.8	36.1
5	36.5	35.9	35.9	36.1
6	36.5	35.8	35.7	36.3
7	36.5	36.3	35.8	36.3
8	36.5	35.7	35.7	36.2
9	36.5	35.3	35.9	36.2
10	36.5	36.3	36.0	36.2
Min [°C]	36.4	35.3	35.7	36.1
Max [°C]	36.5	36.4	36.0	36.3
Max–Min difference [°C]	0.1	1.1	0.3	0.2
Mean [°C]	36.48	36.00	35.81	36.23
GT–Mean difference [°C]	−0.28	0.20	0.39	−0.03

**Table A2:** Intra- and inter-rater reliability experiment. *Subject 2* body temperature data.

**Room temperature** **[°C]**		19.8		
**Room humidity**		53%		
**Replicate 1**				
**Body temperature GT [°C]**		35.9		
**Number**	**T1 [°C]**	**T2 [°C]**	**T3 [°C]**	**T4 [°C]**
1	35.7	34.7	35.8	36.2
2	35.9	35.4	35.6	36.2
3	36.0	35.1	35.8	36.1
4	35.9	36.0	35.6	36.2
5	36.0	35.5	35.8	36.2
6	36.0	35.7	35.8	36.2
7	35.9	35.5	35.8	36.1
8	36.0	35.7	35.8	36.3
9	35.9	36.1	35.8	36.3
10	36.1	35.6	35.7	36.3
Min [°C]	35.7	34.7	35.6	36.1
Max [°C]	36.1	36.1	35.8	36.3
Max–Min difference [°C]	0.4	1.4	0.2	0.2
Mean [°C]	35.94	35.53	35.75	36.21
GT–Mean difference [°C]	−0.04	0.37	0.15	−0.31
**Replicate 2**				
**Body temperature GT [°C]**		36.1		
**Number**	**T1 [°C]**	**T2 [°C]**	**T3 [°C]**	**T4 [°C]**
1	36.0	35.7	35.7	36.3
2	36.1	35.1	35.6	36.1
3	36.1	35.4	35.7	36.1
4	36.1	35.6	35.7	36.3
5	36.1	35.6	35.8	36.2
6	36.1	35.7	35.8	36.2
7	36.2	36.3	35.8	36.2
8	36.3	36.0	35.6	36.2
9	36.1	35.9	35.8	36.2
10	36.0	35.6	35.7	36.3
Min [°C]	36.0	35.1	35.6	36.1
Max [°C]	36.3	36.3	35.8	36.3
Max–Min difference [°C]	0.3	1.2	0.2	0.2
Mean [°C]	36.11	35.69	35.72	36.21
GT–Mean difference [°C]	−0.01	0.41	0.38	−0.11
**Replicate 3**				
**Body temperature GT [°C]**		36.1		
**Number**	**T1 [°C]**	**T2 [°C]**	**T3 [°C]**	**T4 [°C]**
1	36.0	35.5	35.8	36.3
2	36.2	36.0	35.8	36.3
3	36.1	35.2	35.7	36.1
4	36.2	36.1	35.8	36.1
5	36.3	36.2	35.7	36.3
6	36.3	36.2	35.7	36.3
7	36.2	36.2	35.8	36.3
8	36.2	36.1	35.9	36.2
9	36.3	35.3	35.7	36.2
10	36.2	35.1	35.9	36.2
Min [°C]	36.0	35.1	35.7	36.1
Max [°C]	36.3	36.2	35.9	36.3
Max–Min difference [°C]	0.3	1.1	0.2	0.2
Mean [°C]	36.20	35.79	35.78	36.23
GT–Mean difference [°C]	−0.10	0.31	0.32	−0.13

**Table A3:** Intra- and inter-rater reliability experiment. *Subject 3* body temperature data.

**Room temperature** **[°C]**		20.0		
**Room humidity**		51%		
**Replicate 1**				
**Body temperature GT [°C]**		36.4		
**Number**	**T1 [°C]**	**T2 [°C]**	**T3 [°C]**	**T4 [°C]**
1	36.1	36.3	35.8	36.4
2	36.2	36.2	35.9	36.4
3	36.4	36.1	36.0	36.4
4	36.3	35.7	35.9	36.4
5	36.2	36.4	35.8	36.4
6	36.4	36.3	35.9	36.4
7	36.3	36.2	35.9	36.5
8	36.3	36.3	36.0	36.6
9	36.1	36.2	36.0	36.6
10	36.3	35.9	35.9	36.5
Min [°C]	36.1	35.7	35.8	36.4
Max [°C]	36.4	36.4	36.0	36.6
Max–Min difference [°C]	0.3	0.7	0.2	0.2
Mean [°C]	36.26	36.16	35.91	36.46
GT–Mean difference [°C]	0.14	0.24	0.49	−0.06
**Replicate 2**				
**Body temperature GT [°C]**		36.3		
**Number**	**T1 [°C]**	**T2 [°C]**	**T3 [°C]**	**T4 [°C]**
1	36.4	35.9	36.0	36.5
2	36.4	36.3	36.0	36.5
3	36.5	36.2	36.0	36.5
4	36.2	36.1	35.9	36.5
5	36.0	36.4	36.0	36.5
6	36.0	36.1	36.0	36.5
7	36.0	36.0	36.1	36.6
8	36.4	36.0	36.1	36.6
9	36.5	35.8	35.9	36.5
10	36.1	36.0	36.0	36.4
Min [°C]	36.0	35.8	35.9	36.4
Max [°C]	36.5	36.4	36.1	36.6
Max–Min difference [°C]	0.5	0.6	0.2	0.2
Mean [°C]	36.25	36.08	36.00	36.51
GT–Mean difference [°C]	0.05	0.22	0.30	−0.21
**Replicate 3**				
**Body temperature GT [°C]**		36.3		
**Number**	**T1 [°C]**	**T2 [°C]**	**T3 [°C]**	**T4 [°C]**
1	36.0	36.0	36.0	36.4
2	36.2	35.8	36.0	36.4
3	36.3	36.0	35.9	36.5
4	36.0	36.0	36.0	36.4
5	36.1	36.2	36.0	36.4
6	36.4	36.4	36.0	36.5
7	36.5	36.0	36.0	36.4
8	36.1	35.7	36.0	36.4
9	36.4	35.5	36.0	36.4
10	36.4	36.0	35.9	36.4
Min [°C]	36.0	35.5	35.9	36.4
Max [°C]	36.5	36.4	36.0	36.5
Max–Min difference [°C]	0.5	0.9	0.1	0.1
Mean [°C]	36.24	35.96	35.98	36.42
GT–Mean difference [°C]	0.06	0.34	0.32	−0.12

**Table A4:** Body–device distance experiment. *Subject 1* body temperature data.

**Room temperature** **[°C]**		20.1		
**Room humidity**		53%		
**Body temperature GT [°C]**		36.3		
**Distance [cm]–number**	**T1 [°C]**	**T2 [°C]**	**T3 [°C]**	**T4 [°C]**
1 cm–1	36.5	36.4	36.1	36.3
1 cm–2	36.5	36.4	36.1	36.3
1 cm–3	36.5	36.4	36.1	36.2
1 cm–4	36.5	36.4	36.1	36.3
1 cm–5	36.5	36.4	36.1	36.3
2 cm–1	36.6	36.3	36.2	36.2
2 cm–2	36.6	36.3	36.1	36.3
2 cm–3	36.6	36.3	36.2	36.3
2 cm–4	36.5	36.3	36.1	36.3
2 cm–5	36.6	36.3	36.2	36.3
3 cm–1	36.6	36.3	36.1	36.3
3 cm–2	36.6	36.3	36.2	36.3
3 cm–3	36.6	36.3	36.1	36.3
3 cm–4	36.6	36.3	36.2	36.3
3 cm–5	36.7	36.3	36.2	36.3
4 cm–1	36.7	36.3	36.2	36.3
4 cm–2	36.6	36.3	36.2	36.3
4 cm–3	36.6	36.3	36.2	36.3
4 cm–4	36.6	36.3	36.2	36.3
4 cm–5	36.7	36.3	36.2	36.3
5 cm–1	36.7	36.2	36.2	36.3
5 cm–2	36.7	36.2	36.2	36.3
5 cm–3	36.7	36.2	36.2	36.3
5 cm–4	36.7	36.2	36.2	36.3
5 cm–5	36.7	36.2	36.2	36.3
6 cm–1	36.7	35.7	36.3	36.3
6 cm–2	36.7	35.7	36.2	36.3
6 cm–3	36.7	35.7	36.2	36.3
6 cm–4	36.7	35.7	36.2	36.3
6 cm–5	36.7	35.7	36.2	36.3
7 cm–1	36.7	35.3	36.1	36.3
7 cm–2	36.7	35.2	36.2	36.3
7 cm–3	36.7	35.2	36.1	36.3
7 cm–4	36.7	35.3	36.1	36.3
7 cm–5	36.7	35.3	36.1	36.3
8 cm–1	36.7	34.7	36.2	36.3
8 cm–2	36.7	34.6	36.1	36.3
8 cm–3	36.7	34.6	36.1	36.3
8 cm–4	36.7	34.6	36.2	36.3
8 cm–5	36.7	34.7	36.2	36.3
9 cm–1	36.6	33.9	36.0	36.3
9 cm–2	36.6	33.9	36.1	36.3
9 cm–3	36.6	34.0	35.9	36.3
9 cm–4	36.6	34.0	36.1	36.3
9 cm–5	36.7	34.0	36.0	36.3
10 cm–1	36.6	33.5	36.0	36.3
10 cm–2	36.6	33.5	36.0	36.3
10 cm–3	36.6	33.5	36.0	36.3
10 cm–4	36.6	33.5	36.0	36.3
10 cm–5	36.6	33.5	36.0	36.3
Min [°C]	36.5	33.5	35.9	36.2
Max [°C]	36.7	36.4	36.3	36.3
Max–Min difference [°C]	0.2	2.9	0.4	0.1
Mean [°C]	36.64	35.46	36.13	36.30

**Table A5:** Body–device distance experiment. *Subject 2* body temperature data.

**Room temperature** **[°C]**		20.1		
**Room humidity**		53%		
**Body temperature GT [°C]**		36.3		
**Distance [cm]–number**	**T1 [°C]**	**T2 [°C]**	**T3 [°C]**	**T4 [°C]**
1 cm–1	36.2	36.3	35.9	36.2
1 cm–2	36.2	36.3	35.9	36.2
1 cm–3	36.3	36.3	35.8	36.2
1 cm–4	36.3	36.3	35.8	36.2
1 cm–5	36.4	36.3	35.9	36.2
2 cm–1	36.4	36.3	35.8	36.2
2 cm–2	36.4	36.3	35.8	36.2
2 cm–3	36.4	36.3	35.8	36.2
2 cm–4	36.4	36.3	35.8	36.2
2 cm–5	36.5	36.3	35.8	36.2
3 cm–1	36.4	36.3	35.9	36.2
3 cm–2	36.4	36.3	36.0	36.2
3 cm–3	36.4	36.3	35.9	36.2
3 cm–4	36.5	36.3	35.9	36.2
3 cm–5	36.5	36.3	36.0	36.2
4 cm–1	36.5	35.9	36.0	36.2
4 cm–2	36.5	36.0	36.1	36.2
4 cm–3	36.5	35.9	36.0	36.2
4 cm–4	36.5	35.9	36.0	36.2
4 cm–5	36.5	35.5	36.0	36.2
5 cm–1	36.5	35.5	36.0	36.2
5 cm–2	36.5	35.6	36.0	36.2
5 cm–3	36.5	35.4	35.9	36.2
5 cm–4	36.5	35.5	36.0	36.2
5 cm–5	36.6	34.5	35.9	36.2
6 cm–1	36.6	34.5	35.9	36.2
6 cm–2	36.5	34.6	35.9	36.2
6 cm–3	36.6	34.7	35.9	36.2
6 cm–4	36.6	34.6	35.9	36.2
6 cm–5	36.6	34.7	35.8	36.2
7 cm–1	36.6	34.1	35.8	36.2
7 cm–2	36.5	34.1	35.9	36.2
7 cm–3	36.5	34.1	35.9	36.2
7 cm–4	36.5	34.1	35.8	36.2
7 cm–5	36.5	34.1	35.9	36.2
8 cm–1	36.5	33.8	35.9	36.2
8 cm–2	36.5	33.8	35.9	36.2
8 cm–3	36.6	33.8	35.9	36.2
8 cm–4	36.5	33.8	36.0	36.2
8 cm–5	36.6	33.6	36.0	36.2
9 cm–1	36.5	33.0	35.9	36.2
9 cm–2	36.5	33.0	35.9	36.2
9 cm–3	36.5	33.1	35.9	36.2
9 cm–4	36.5	33.1	36.0	36.2
9 cm–5	36.5	33.1	35.9	36.2
10 cm–1	36.4	33.0	35.9	36.2
10 cm–2	36.4	33.0	35.9	36.2
10 cm–3	36.3	32.9	35.9	36.2
10 cm–4	36.3	33.0	35.9	36.2
10 cm–5	36.4	33.0	35.8	36.2
Min [°C]	36.2	32.9	35.8	36.2
Max [°C]	36.6	36.3	36.1	36.2
Max-Min difference [°C]	0.4	3.4	0.3	0.0
Mean [°C]	36.47	34.86	35.91	36.20

**Table A6:** Body–device distance experiment. *Subject 3* body temperature data.

**Room temperature** **[°C]**		20.0		
**Room humidity**		51%		
**Body temperature GT [°C]**		36.6		
**Distance [cm]–number**	**T1 [°C]**	**T2 [°C]**	**T3 [°C]**	**T4 [°C]**
1 cm–1	36.8	36.5	36.0	36.3
1 cm–2	36.7	36.5	36.1	36.3
1 cm–3	36.8	36.5	36.1	36.3
1 cm–4	36.8	36.5	36.1	36.3
1 cm–5	36.8	36.5	36.1	36.3
2 cm–1	36.9	36.4	36.2	36.3
2 cm–2	36.8	36.4	36.2	36.3
2 cm–3	36.8	36.4	36.2	36.3
2 cm–4	36.8	36.4	36.1	36.3
2 cm–5	36.9	36.4	36.2	36.4
3 cm–1	36.9	36.1	36.2	36.4
3 cm–2	36.9	36.1	36.3	36.4
3 cm–3	36.9	36.1	36.3	36.4
3 cm–4	36.9	36.1	36.2	36.4
3 cm–5	36.9	36.1	36.2	36.4
4 cm–1	36.9	36.0	36.2	36.4
4 cm–2	36.9	35.9	36.3	36.4
4 cm–3	36.9	35.9	36.3	36.4
4 cm–4	37.0	35.9	36.3	36.4
4 cm–5	37.0	35.5	36.3	36.4
5 cm–1	37.0	35.4	36.3	36.4
5 cm–2	37.0	35.4	36.3	36.4
5 cm–3	37.0	35.4	36.3	36.4
5 cm–4	37.0	35.3	36.3	36.4
5 cm–5	37.0	35.3	36.3	36.4
6 cm–1	36.9	34.6	36.3	36.4
6 cm–2	36.9	34.6	36.3	36.4
6 cm–3	36.9	34.6	36.3	36.4
6 cm–4	36.9	34.6	36.2	36.4
6 cm–5	36.9	34.6	36.3	36.4
7 cm–1	36.9	33.9	36.3	36.4
7 cm–2	36.9	33.9	36.3	36.4
7 cm–3	36.9	33.9	36.3	36.4
7 cm–4	36.9	33.8	36.3	36.4
7 cm–5	36.9	33.7	36.3	36.4
8 cm–1	36.9	33.5	36.2	36.4
8 cm–2	36.9	33.5	36.2	36.4
8 cm–3	36.9	33.5	36.3	36.4
8 cm–4	36.9	33.5	36.3	36.4
8 cm–5	36.9	33.5	36.3	36.4
9 cm–1	36.8	33.4	36.2	36.4
9 cm–2	36.8	33.5	36.3	36.4
9 cm–3	36.8	33.3	36.4	36.4
9 cm–4	36.8	33.4	36.4	36.4
9 cm–5	36.8	33.4	36.4	36.4
10 cm–1	36.8	32.6	36.3	36.4
10 cm–2	36.8	32.6	36.3	36.4
10 cm–3	36.8	32.6	36.3	36.4
10 cm–4	36.8	32.7	36.3	36.4
10 cm–5	36.8	32.6	36.3	36.4
Min [°C]	36.7	32.6	36.0	36.3
Max [°C]	37.0	36.5	36.4	36.4
Max-Min difference [°C]	0.3	3.9	0.4	0.1
Mean [°C]	36.88	34.82	36.26	36.38

**Table A7:** Body–device angle experiment. *Subject 1* body temperature data.

**Room temperature** **[°C]**		20.2		
**Room humidity**		53%		
**Body temperature GT [°C]**		36.3		
**Degree [°]–number**	**T1 [°C]**	**T2 [°C]**	**T3 [°C]**	**T4 [°C]**
0°–1	36.2	36.3	35.9	36.2
0°–2	36.2	36.3	36.1	36.2
0°–3	36.2	36.3	36.0	36.2
0°–4	36.3	36.3	36.0	36.2
0°–5	36.3	36.3	36.0	36.2
23°–1	36.1	36.1	36.1	36.2
23°–2	36.2	36.1	36.2	36.2
23°–3	36.1	36.2	36.1	36.2
23°–4	36.1	36.0	36.2	36.2
23°–5	36.2	36.0	36.1	36.2
45°–1	35.9	35.7	36.0	36.2
45°–2	35.9	35.7	36.0	36.2
45°–3	35.9	35.7	36.0	36.2
45°–4	35.9	35.8	36.0	36.2
45°–5	35.9	35.7	36.0	36.2
Min [°C]	35.9	35.7	35.9	36.2
Max [°C]	36.3	36.3	36.2	36.2
Max-Min difference [°C]	0.4	0.6	0.3	0.0
Mean [°C]	36.09	36.03	36.05	36.20

**Table A8:** Body–device angle experiment. *Subject 2* body temperature data.

**Room temperature** **[°C]**		20.2		
**Room humidity**		50%		
**Body temperature GT [°C]**		36.3		
**Degree [°]–number**	**T1 [°C]**	**T2 [°C]**	**T3 [°C]**	**T4 [°C]**
0°–1	36.1	36.0	35.8	36.3
0°–2	36.1	36.1	35.7	36.3
0°–3	36.2	36.0	35.7	36.3
0°–4	36.2	36.1	35.8	36.3
0°–5	36.2	36.1	35.8	36.3
23°–1	36.3	35.6	35.8	36.3
23°–2	36.2	35.4	35.8	36.3
23°–3	36.2	35.4	35.8	36.3
23°–4	36.2	35.4	35.9	36.3
23°–5	36.2	35.3	35.8	36.3
45°–1	36.1	34.9	35.8	36.3
45°–2	36.1	34.8	35.8	36.3
45°–3	36.1	34.8	35.8	36.3
45°–4	36.1	34.8	35.8	36.3
45°–5	36.1	34.9	35.8	36.3
Min [°C]	36.1	34.8	35.7	36.3
Max [°C]	36.3	36.1	35.9	36.3
Max-Min difference [°C]	0.2	1.3	0.2	0.0
Mean [°C]	36.16	35.44	35.79	36.30

**Table A9:** Body–device angle experiment. *Subject 3* body temperature data.

**Room temperature** **[°C]**		20.0		
**Room humidity**		51%		
**Body temperature GT [°C]**		36.6		
**Degree [°]–number**	**T1 [°C]**	**T2 [°C]**	**T3 [°C]**	**T4 [°C]**
0°–1	36.7	36.3	36.2	36.6
0°–2	36.7	36.3	36.2	36.6
0°–3	36.7	36.3	36.2	36.5
0°–4	36.7	36.3	36.2	36.6
0°–5	36.7	36.3	36.2	36.6
23°–1	36.5	36.0	36.1	36.6
23°–2	36.5	36.0	36.2	36.6
23°–3	36.5	36.0	36.2	36.6
23°–4	36.5	35.9	36.2	36.6
23°–5	36.5	36.0	36.1	36.6
45°–1	36.5	35.3	36.1	36.6
45°–2	36.4	35.2	36.1	36.6
45°–3	36.4	35.2	36.1	36.6
45°–4	36.4	35.3	36.1	36.6
45°–5	36.5	35.2	36.2	36.6
Min [°C]	35.9	35.7	35.9	36.2
Max [°C]	36.3	36.3	36.2	36.2
Max-Min difference [°C]	0.4	0.6	0.3	0.0
Mean [°C]	36.09	36.03	36.05	36.20

**Table A10:** Light influence experiment. *Subject 1* body temperature data.

**Room temperature** **[°C]**		20.4		
**Room humidity**		50%		
**Body temperature GT [°C]**		36.3		
**Light setting–number**	**T1 [°C]**	**T2 [°C]**	**T3 [°C]**	**T4 [°C]**
*I*-1	36.5	36.3	36.4	36.2
*I*-2	36.5	36.3	36.4	36.2
*I*-3	36.5	36.3	36.4	36.2
*I*-4	36.5	36.3	36.4	36.2
*I*-5	36.5	36.3	36.4	36.2
*II*-1	36.6	36.1	36.4	36.2
*II*-2	36.5	36.1	36.5	36.2
*II*-3	36.5	36.2	36.5	36.2
*II*-4	36.5	36.0	36.5	36.2
*II*-5	36.5	36.0	36.5	36.2
*III*-1	36.5	35.9	36.6	36.2
*III*-2	36.5	35.9	36.6	36.2
*III*-3	36.5	36.4	36.7	36.2
*III*-4	36.6	36.4	36.6	36.2
*III*-5	36.6	36.4	36.5	36.2
Min [°C]	36.5	35.9	36.4	36.2
Max [°C]	36.6	36.4	36.7	36.2
Max-Min difference [°C]	0.1	0.5	0.3	0.0
Mean [°C]	36.52	36.19	36.49	36.20

**Table A11:** Light influence experiment. *Subject 2* body temperature data.

**Room temperature** **[°C]**		20.4		
**Room humidity**		50%		
**Body temperature GT [°C]**		36.3		
**Light setting–number**	**T1 [°C]**	**T2 [°C]**	**T3 [°C]**	**T4 [°C]**
*I*–1	36.3	36.1	36.0	36.3
*I*–2	36.3	36.1	36.0	36.3
*I*–3	36.3	36.1	36.0	36.2
*I*–4	36.4	36.2	36.0	36.3
*I*–5	36.3	36.2	36.0	36.2
*II*–1	36.4	36.2	36.0	36.3
*II*–2	36.4	36.1	36.0	36.3
*II*–3	36.4	36.2	36.0	36.3
*II*–4	36.5	36.2	36.0	36.3
*II*–5	36.5	36.1	36.0	36.3
*III*–1	36.5	36.2	36.1	36.3
*III*–2	36.6	36.2	36.0	36.3
*III*–3	36.6	36.2	36.1	36.3
*III*–4	36.7	36.2	36.1	36.3
*III*–5	36.7	36.2	36.0	36.3
Min [°C]	36.3	36.1	36.0	36.2
Max [°C]	36.7	36.2	36.1	36.3
Max-Min difference [°C]	0.4	0.1	0.1	0.1
Mean [°C]	36.46	36.17	36.02	36.29

**Table A12:** Light influence experiment. *Subject 3* body temperature data.

**Room temperature** **[°C]**		20.0		
**Room humidity**		51%		
**Body temperature GT [°C]**		36.3		
**Light setting–number**	**T1 [°C]**	**T2 [°C]**	**T3 [°C]**	**T4 [°C]**
*I*–1	36.7	36.3	36.3	36.5
*I*–2	36.8	36.3	36.3	36.5
*I*–3	36.7	36.3	36.3	36.5
*I*–4	36.7	36.3	36.3	36.5
*I*–5	36.7	36.3	36.3	36.5
*II*–1	36.8	36.1	36.3	36.5
*II*–2	36.8	36.1	36.3	36.5
*II*–3	36.8	36.1	36.3	36.5
*II*–4	36.8	36.2	36.4	36.5
*II*–5	36.8	36.3	36.4	36.5
*III*–1	36.8	36.0	36.4	36.5
*III*–2	36.9	36.2	36.4	36.5
*III*–3	36.8	36.1	36.4	36.5
*III*–4	36.9	36.1	36.4	36.5
*III*–5	36.9	36.1	36.4	36.5
Min [°C]	36.7	36.0	36.3	36.5
Max [°C]	36.9	36.3	36.4	36.5
Max-Min difference [°C]	0.2	0.3	0.1	0.0
Mean [°C]	36.8	36.2	36.3	36.5
